# A novel and highly efficient Zr-containing catalyst supported by biomass-derived sodium carboxymethyl cellulose for hydrogenation of furfural

**DOI:** 10.3389/fchem.2022.966270

**Published:** 2022-07-22

**Authors:** Jianxiu Hao, Yafang Zhang, Tianyuan Zhang, Huacong Zhou, Quansheng Liu, Keduan Zhi, Na Li, Runxia He

**Affiliations:** College of Chemical Engineering, Inner Mongolia University of Technology, Hohhot, NM, China

**Keywords:** sodium carboxymethyl cellulose, furfural, hydrogenation, catalyst, biomass

## Abstract

Functional use of biomass based on its structural properties is an efficient approach for the valuable utilization of biomass resources. In this work, carboxymethyl cellulose zirconium-based catalyst (Zr-CMC) was constructed by the coordination between the carboxylic groups in sodium carboxymethyl cellulose (CMC-Na) with transition metal Zr^4+^. The prepared catalyst was applied into the synthesis of furfuryl alcohol (FAL) by catalytic transfer hydrogenation of biomass-derived furfural (FF) using isopropanol as hydrogen donor. Both the preparation conditions and the reaction conditions of Zr-CMC catalyst were investigated and optimized. The results showed that Zr-CMC was efficient for the reaction with the FF conversion, FAL yield and selectivity reaching to 92.5%, 91.5 %, and 99.0%, respectively, under the mild conditions (90°C). Meanwhile, the Zr-CMC catalyst could be reused at least for five times without obvious decrease in efficiency, indicating the catalyst had excellent stability. With the advantages of sustainable raw materials, high efficiency, and excellent stability, the prepared catalyst is potential for application in the field of biomass conversion.

## 1 Introduction

As an important carbon source on Earth, biomass is abundant, renewable and can produce fuel and high-value chemicals through transformation. It is expected to replace or partially replace traditional fossil resources and reduce the dependence on fossil resources for human. The high-value utilization of biomass resources can improve the economy and utilization efficiency of carbon resources in biomass refining process, and the production of high-value chemicals from biomass or its derivatives is an effective way to improve the utilization efficiency of biomass resources (Li et al., 2016a). Among the various platform compounds from biomass, furfural is a valuable chemical derived from pentosan by hydrolyzing dehydration and cyclization reaction. Furfural can be used to produce valuable chemicals such as furfuryl alcohol (FA), cyclopentanone (CLA), 2-methylfuran (2-MF) etcetera through hydrogenation (Wang et al., 2018a; Pirmoradi et al., 2020; Byun and Lee, 2021). Furfuryl alcohol is an important organic chemical raw material, mainly used in the production of furfural resin, furfuryl alcohol-ural resin, phenolic resin and so on. It is also used to prepare fruit acid, plasticizer, solvent and rocket fuel. In addition, in the fields of dye, synthetic fiber, rubber, pesticides and casting and other industrial sectors, furfuryl alcohol is also widely used (Wang et al., 2018b; Campisi et al., 2020; Lee et al., 2020; Qiu et al., 2020).

For the hydrogenation of furfural to furfuryl alcohol, homogeneous catalysts are usually used, but the homogeneous catalysts are not easy to be recovered in the reaction system (Pasini et al., 2014; Panagiotopoulou et al., 2015). Meanwhile, the hydrogenation of furfural to furfural is mainly dependent on copper chromate and expensive precious metal catalyst (Li et al., 2022). Therefore, finding cheap and efficient heterogeneous catalyst is the key for the hydrogenation of furfural to furfuryl alcohol. With the development of heterogeneous catalytic technology, a variety of mono-metal based catalysts such as Zr, Cu, Ru, Ir, Co, Fe, Pd, Pt, etc. (Pang et al., 2014; Gong et al., 2019; Li et al., 2020; Qiu et al., 2020; Sohrabi et al., 2021; Valdebenito et al., 2021) and polymetallic catalysts such as Pt/Ge (Merlo et al., 2011), Cu/Fe (Yan and Chen, 2014), Cu/Mg/Al, etc. (Chen et al., 2018) have been developed, and the catalytic hydrogenation of furfural to alcohols or esters using hydrogen as hydrogen source was also reported (Mariscal et al., 2016). Although the hydrogenation efficiency of carbonyl compounds is improved by using the precious metals and gaseous hydrogen, the high cost of precious metals and the potential safety issue of gaseous hydrogen hinder the large-scale application of many catalysts.

Meerwein-Ponndorf-Verley (MPV) reduction is a common reaction for the catalytic hydrogenation of biomass derived aldehydes or carbonyls with secondary alcohols as hydrogen donors (Luo et al., 2014). Using alcohols as hydrogen source can not only avoid safety risks caused by high-pressure hydrogen, but also improve the economy of the hydrogenation of carbonyl compounds with relatively mild reaction conditions. The secondary alcohols play the role of both hydrogen donor and the reaction solvent for the catalytic hydrogenation of carbonyl compounds to produce high-value chemicals (Wright and Palkovits, 2012). The preparation of furfuryl alcohol by MPV reduction of furfural catalyzed by different heterogeneous metal catalysts (Han et al., 2015; Li et al., 2022) has been reported. Metal ion-organic ligand hybrid MPV catalysts are commonly used for the MPV reduction of furfural. The Zr-based catalysts such as UiO-66 (Qiu et al., 2020), Zr-DM (Hao et al., 2021), Zr-HAs (Sha et al., 2017), Zr-RSL (Hao et al., 2019) and Zr/Fe (Gu et al., 2020) showed good catalytic activity in the catalytic hydrogenation reaction using alcohols as hydrogen donors. The selection of ligands is important for the construction of metal ion-organic ligand hybrid MPV catalysts. Besides the pure organic carboxylic acids, the natural macromolecules rich in organic carboxylic groups are also used in the construction of the MPV catalysts. In our previous work, the lignite derived humic acids and even lignite were used to construct Zr-containing MPV catalysts, and the prepared catalysts were proved to be highly efficient for the MPV reduction of various carbonyl compounds (Sha et al., 2017; Hao et al., 2019; Hao et al., 2021). Natural compounds rich in acidic carboxyl functional groups are natural functional molecules widely existing in nature (Luo et al., 2015). With the advantages of wide sources, low cost, renewable, etc., natural compounds are widely used in biological materials, catalytic materials, and other fields (Sahiner et al., 2015). Sodium carboxymethyl cellulose (CMC-Na) is a common anionic polymer compound derived from natural cellulose with abundant carboxylate groups in its structure. CMC-Na can be used as a ligand or carrier to construct a variety of metal catalysts. For example, carboxymethyl cellulose palladium (II) (CMC-PdII) catalyst was prepared by ion exchange between PdCl_2_ aqueous solution and CMC-Na aqueous solution to catalyze the Suzuki-Miyaura and Mizoroki-heck coupling reaction (Xiao et al., 2015). The Fe_3_O_4_/CMC composite material was prepared by coprecipitating method with iron salt and CMC-Na. The Ni(0)-CMC-Na catalyst was obtained using the CMC-Na aqueous solution and the precursor NiCl_2_·6H_2_O, and the catalyst showed high efficiency for the hydrogenation of a variety of functionalized olefins at room temperature and moderate hydrogen pressures (Harrad et al., 2011). Based on the reported results, it is reasonable to speculate that CMC-Na could be applied into the construction of metal ion-organic ligand MPV catalyst for the conversion of furfural. However, to the best of our knowledge, there has been no report on the above idea.

In this work, a novel Zr-CMC catalyst was constructed using CMC-Na and Zr precursor as raw material *via* the interaction between the carboxylate groups in CMC-Na and transition metal Zr^4+^. The prepared catalyst was used for the catalytic transfer hydrogenation of furfural to furfuryl alcohol using isopropanol as hydrogen donor. The effects of the preparation conditions and the reaction conditions on the performance of the catalyst were studied. The structure and the reusability of the catalyst were investigated. This work can provide potential route for the exploration of efficient, cheap and green catalysts for biomass conversion and the efficient utilization of CMC biomass resources.

## 2 Experimental

### 2.1 Materials

Furfural (FF, 99%) (Furfural used in this work was freshly purchased and stored in the refrigerator (4°C). Before use, furfural was analyzed by GC to check its purity), furfuryl alcohol (FA, 98%) and ZrOCl_2_·8H_2_O (AR) were provided by J&K Scientific Ltd. Isopropanol (AR), ethanol (AR), decane (AR) and other chemicals were obtained from Beijing Institute of Chemical Reagent. The sodium carboxymethyl cellulose was purchased from Tianjin Guangfu Fine Chemical Research Institute.

### 2.2 Catalyst preparation

Zr-based catalyst was prepared using sodium carboxymethyl cellulose (CMC-Na) and ZrOCl_2_·8H_2_O as the raw materials. In order to determine the appropriate ratio of sodium carboxymethyl cellulose and zirconium oxychloride octahydrate, a series of catalysts with different ratios were prepared, and their catalytic activity for hydrogenation of furfural was investigated under the same conditions, and then the appropriate ratio was selected. The mass ratios of sodium carboxymethyl cellulose and ZrOCl_2_·8H_2_O were 1:0.5, 1:1, 1:2, and 1:3, respectively.

**FIGURE 1:**
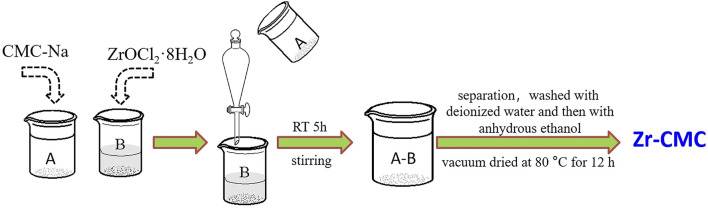
The preparation scheme diagram of the Zr-CMC catalyst.

Typical procedures were as follows (Figure 1): a certain amount of sodium carboxymethyl cellulose dissolved in 150 ml deionized water (solution A), heated and stirred in 60°C water bath to make it fully dissolved into transparent gel. At the same time, a certain amount of ZrOCl_2_·8H_2_O was proportionally weighed and dissolved in 50 ml deionized water (solution B). Pour the solution A into the separating funnel and slowly add it to the solution B, and stir the reaction system at room temperature for 5 h after dropping. The solution was then pumped and filtered or centrifuged (pH = 2–3), and washed with deionized water for at least seven times until pH neutralization. Rinse twice with anhydrous ethanol to remove the deionized water. Finally, the obtained solids were vacuum dried at 80°C for 12 h. The dried solids were ground thoroughly into powder form for subsequent use, denoted as Zr-CMC.

### 2.3 Catalyst characterization

Scanning electron microscopy (SEM) measurements were performed on a HITACHI SU8220 scanning electron microscope operated at 20 kV. Transmission electron microscopy (TEM) images were obtained using a TEM JEOL-1011 with an accelerating voltage of 120 kV. X-ray diffraction (XRD) was carried out *via* an XD8 Advance-Bruker AXS X-ray diffractometer using Cu-Kα radiation (*λ* = 0.1543 nm) and Ni filter scanning at two per minute ranging from 5° to 90°. The tube voltage was 40 kV, and the current was 40 mA. Fourier transform-infrared spectra (FT-IR) were obtained using a PerkinElmer spectrometer. The thermogravimetric (TG) analysis of the catalyst was performed using a thermogravimetric analysis system (Diamond TG/DTA6300, PerkinElmer Instruments) under N_2_ atmosphere at the heating rate of 10°C min^−1^. The specific surface area was calculated by the BET method, and mesopore volume was derived from the adsorption isotherm according to the Barrett−Joyner−Halenda (BJH) model. All calculations were based on the adsorption isothermals.

### 2.4 Reaction

#### 2.4.1 Catalytic transfer hydrogenation of furfural over Zr-CMC

All the hydrogenation of furfural to furfuryl alcohol in this experiment was carried out in a stainless steel reactor. In the lining of the reaction kettle (15 ml), accurate weighing of reactant furfural (1 mmol) and isopropanol (5 ml) as hydrogen source and reaction solvent, as well as a certain amount of catalyst and decane as internal standard, using atmospheric pressure operation, the reaction kettle is sealed and placed in an oil bath with constant temperature heating magnetic stirring. After the reaction, remove the reactor quickly from the oil bath and place it in the mixture of ice water for rapid cooling. After cooling, the reaction solution is separated from the solid catalyst by centrifugation. The residue of reaction product and substrate can be quantitatively analyzed by gas chromatograph (TECHCOMP GC7900). The specific conditions of gas chromatography were as follows: hydrogen flame ion detector (FID), nitrogen as carrier gas, inlet temperature of 40°C, detection temperature of 230°C, set the heating program for furfural column temperature program, using area internal standard method for quantitative analysis of the reactant furfural and target product furfuryl alcohol, using decane as internal standard.

#### 2.4.2 Catalyst heterogeneity and recycles

In order to check the heterogeneity of the catalysts, the solid catalysts were removed from the reaction mixture after reaction for certain time, and the supernatant was allowed to react to see if the product yield could further increase with the absence of the solid catalysts. In the reusability experiments, the catalyst was separated by centrifugation, washed with fresh isopropanol for three times, and then reused for the next run without further treatments.

## 3 Results and discussion

### 3.1 Studies of the preparation conditions of Zr-CMC catalyst

Table 1 showed the comparison of the activity of the catalysts prepared in different proportions. The results showed that with the increase of ZrOCl_2_·8H_2_O content, the conversion of furfural, the yield of furfuryl alcohol and the selectivity of the reaction increased gradually. When the mass ratio of CMC-Na to ZrOCl_2_·8H_2_O was 1:2, the activity of the prepared catalyst reached the highest, possibly because the carboxylic acid groups in CMC-Na and Zr^4+^ were completely combined at this ratio. With the further increase of the mass of ZrOCl_2_·8H_2_O, the catalytic performance decreases, indicating that there is no excess carboxylic acid group in CMC-Na for the coordination of Zr^4+^. It may be due to that when the mass ratio of CMC-Na to ZrOCl_2_.8H_2_O is 1:3, the acidity of the solution increased, which may affect the coordination reaction between zirconium precursor and carboxylic acid in CMC-Na, leading to the low content of Zr in the catalyst. Besides, the precipitation amount obtained under this condition decreases. Therefore, from viewpoint of catalyst activity and the utilization efficiency of Zr precursor and CMC-Na, the best mass ratio of CMC-Na and ZrOCl_2_·8H_2_O in the preparation process is 1:2.

**TABLE 1:** Performances of Zr-CMC catalysts prepared under different mass ratios of CMC-Na to ZrOCl_2_·8H_2_O.

Entry	Mass ratio of CMC-Na:ZrOCl_2_·8H_2_O	Yield (%)	Conv. (%)	Sel. (%)
1	1:0.5	16.7	20.8	80.6
2	1:1	37.5	44.0	85.1
3	1:2	78.7	79.5	99.0
4	1:3	40.8	48.6	83.9

Reaction conditions: Furfural 1 mmol, isopropanol 5 ml, catalyst dosage 200 mg, reaction temperature 80°C, and reaction time 3 h.

### 3.2 Structure characterization of the Zr-CMC catalyst

**FIGURE 2:**
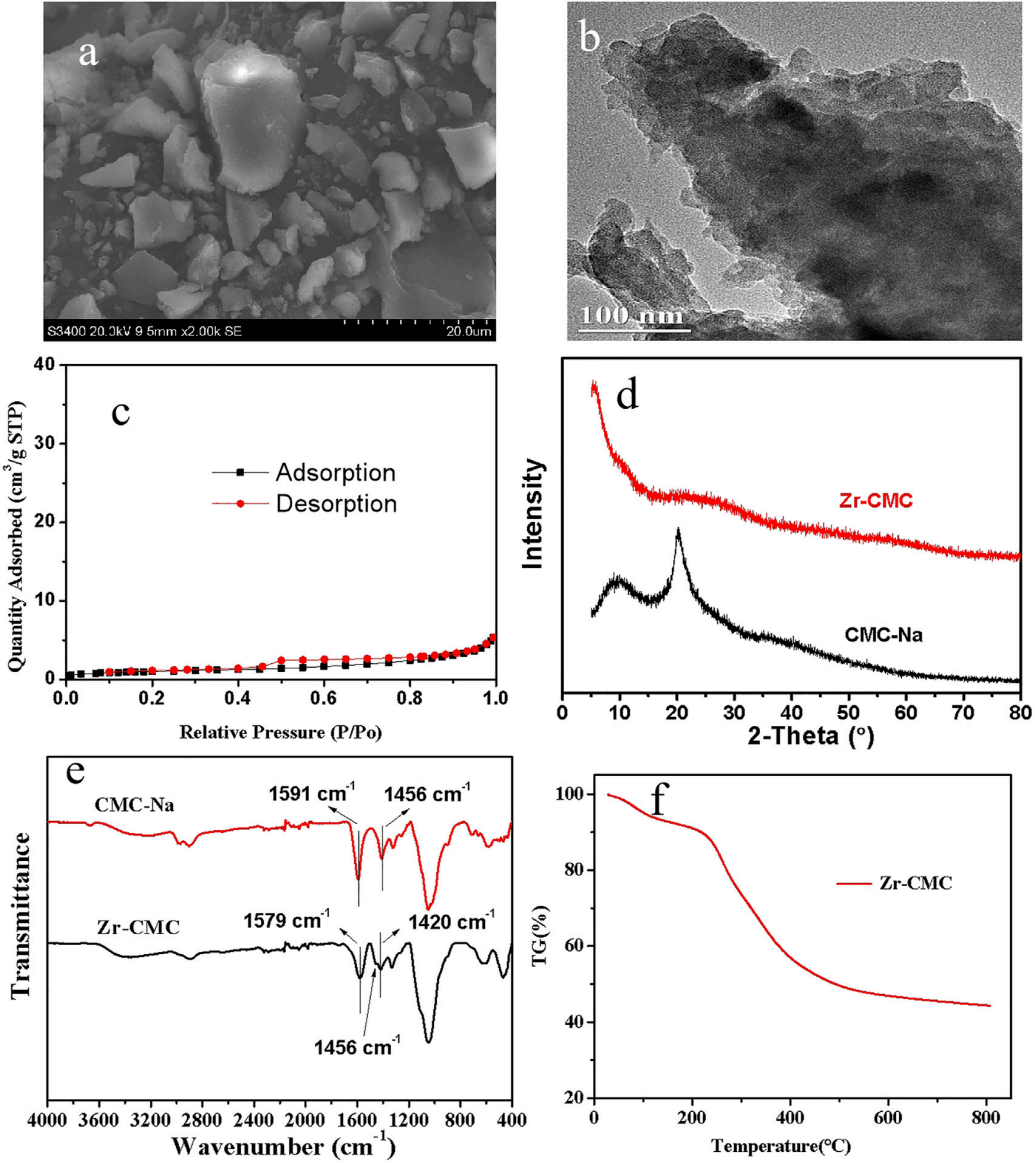
Characterization of the as-prepared Zr-CMC catalyst by SEM **(A)**, TEM **(B)**, N_2_ adsorption-desorption **(C)**, XRD pattern **(D)**, FTIR spectra **(E)** and TG analysis **(F)**.

In order to better understand the structure and morphology characteristics of the prepared Zr-CMC catalyst, SEM, TEM, XRD, FTIR, TG, and BET were used to characterize it. Meanwhile, CMC-Na was also characterized by XRD, FTIR, and TG, and compared with the prepared Zr-CMC catalyst in structure. Firstly, the Zr content in the optimal catalyst was analyzed by ICP-OES, and the result showed that the Zr content in Zr-CMC (1:2) catalyst was around 20.5 wt%. As given in Figure 2, SEM and TEM showed that the Zr-CMC catalyst was composed of particles with no uniform shapes (Figures 2A,B). N_2_ adsorption/desorption test showed that the Zr-CMC catalyst prepared was nonporous (Figure 2C), and the BET surface area was around 3 m^2^/g. The low specific surface area may be caused by the dense structures formed by CMC-Na with Zr precursor and how to improve the surface area is under studying in our group. A strong X-ray diffraction peak was observed for the pure CNC-Na at about 21°, while a weak bulge peak was observed for the Zr-CMC catalyst at 20–30°, indicating that the prepared catalyst had no specific X-ray diffraction peak and had an amorphous structure (Figure 2D). The FTIR spectra of CMC-Na and the Zr-CMC catalyst were compared in Figure 2E. The FTIR spectra of CMC-Na showed that there were asymmetric and symmetric stretching vibration absorption peaks of the carboxyl functional groups at 1,591 and 1,456 cm^−1^, respectively. In the FTIR spectra of Zr-CMC catalyst, the asymmetric and symmetric stretching vibration absorption peaks of the carboxyl functional groups were at 1,579 and 1,420 cm^−1^, respectively. Compared with CMC-Na, the difference between the asymmetric and symmetric stretching vibration absorption peaks of the carboxyl functional groups in Zr-CMC catalyst changes from 135 cm^−1^ to 159 cm^−1^, and a new vibration absorption peak appears at 1,456 cm^−1^. The above results indicate that zirconium is successfully combined with carboxyl functional groups (Song et al., 2015a). As shown in the figure (Figure 2F) for TG analysis, the prepared Zr-CMC catalyst begins to decompose at 300°C, while the reaction temperature is below 100°C. Therefore, the Zr-CMC catalyst is stable under the reaction temperature (Peng et al., 2012).

### 3.3 Studies of the reaction conditions

**FIGURE 3:**
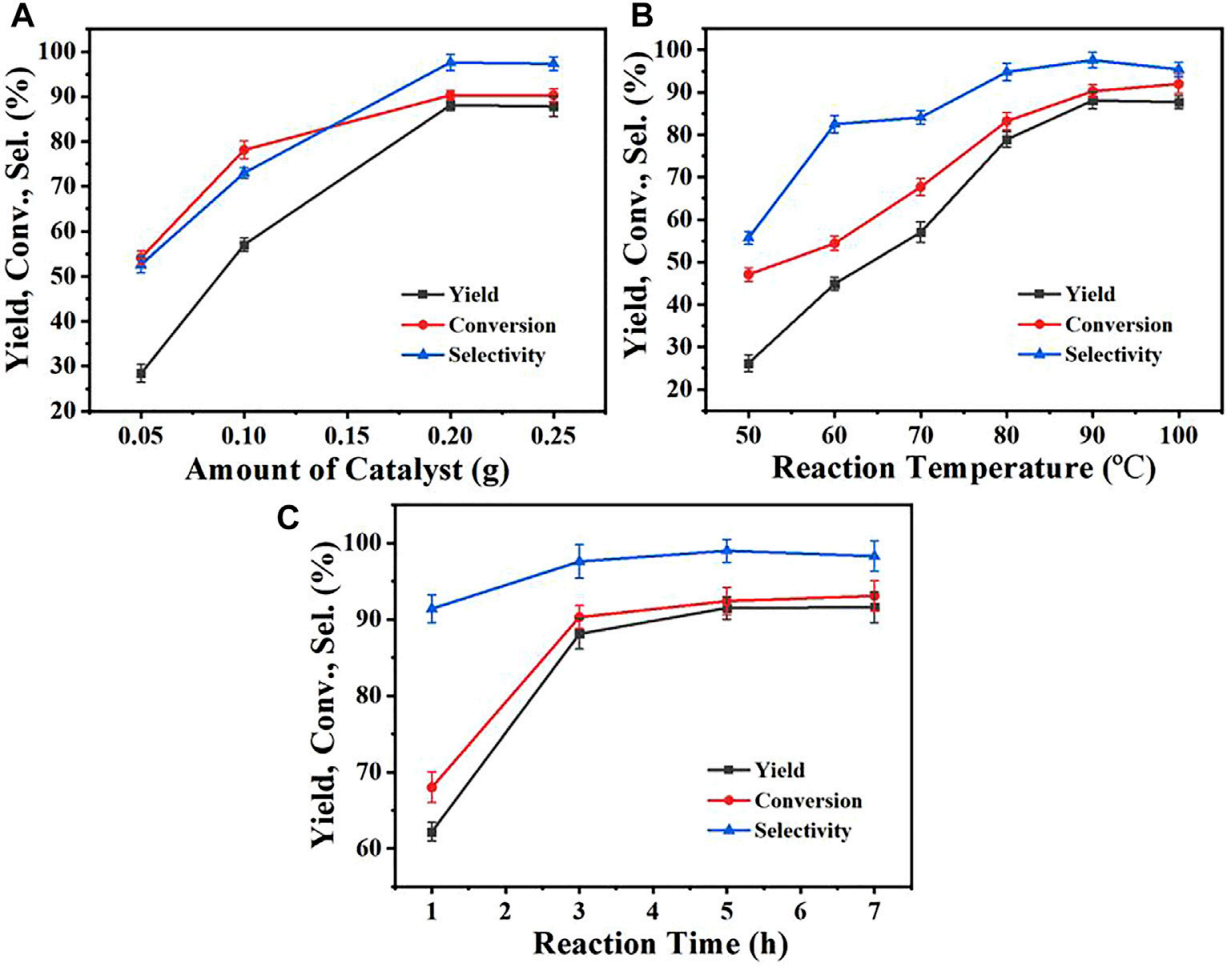
The influences of reaction parameters on the catalysts **(A)** Effect of the Zr-CMC catalyst dosage. **(B)** Effect of the reaction temperature. **(C)** Effect of the reaction time. Reaction conditions: Furfural (1 mmol), isopropanol (5 ml). **(A)** 90°C, 3 h **(B)** Catalyst 0.2 g, 3 h **(C)** Catalyst 0.2 g, 90°C.

The important parameters influencing the performances of the catalysts were investigated, including the catalyst dosage, reaction temperature, and reaction time. Figure 2 showed the performances of the Zr-CMC catalyst under different conditions. As could be seen from Figure 3A, the conversion of furfural and the yield of furfuryl alcohol increased continually with the increasing of the catalyst dosage from 0.05 to 0.2 g. Under the catalyst dosage of 0.2 g, the conversion, yield and selectivity were 87.9%, 96.3 %, and 90.9%, respectively. However, the furfural conversion, furfuryl alcohol yield and reaction selectivity did not increase further when the amount of catalyst was increased to 0.25 g, which may be because the excessive amount of catalyst affected the mass transfer of catalyst in the reaction system. The above results indicate that 0.2 g dosage may be the appropriate catalyst dosage under the current reaction conditions, which can be used for further experimental studies. The effect of reaction temperature was shown in Figure 3B, and it was obvious that the reaction temperature had a significant influence on the performance of the Zr-CMC catalyst. Before 90°C, the furfural conversion and furfural alcohol yield increased significantly with the increase of temperature, reaching 90.9 % and 87.9%, respectively, under 90°C. However, as the temperature continued to rise, although the conversion continued to increase at a slower rate, the yield and selectivity declined slightly, presumably due to the formation of some by-products, which could be proved by the deep color of the reaction system under 100°C. Therefore, subsequent investigations were conducted at 90°C as the suitable temperature under the present reaction conditions. Under the above reaction conditions, the time-profile of the reaction was studied, as shown in Figure 3C. The conversion of furfural and the yield of furfuryl alcohol increased significantly when the reaction time increased from 0.5 to 3 h. As the reaction time was extended to 6 h, the conversion and selectivity of the reactant increased slightly, which may be due to that the extension of the reaction time led to the formation of the condensation by-products of alcohol and aldehyde. This could be proved by the presence of some weak and unknown peaks during GC detect.

After studying the reaction condition, the performance of the prepared Zr-CMC catalyst was compared with other common Zr-based catalysts in the literatures (Table 2). The results showed that the activity of the prepared catalyst was comparable with many other catalysts under similar reaction conditions. The performance of the Zr-CMC catalyst was even superior to some catalyst in aspects of selectivity and TOF. Besides the high activity, the Zr-CMC catalyst possessed the advantages of biomass-derived and renewable raw ligands during preparation, making the Zr-CMC catalyst promising for potential application.

**TABLE 2:** Comparison of the prepared Zr-CMC catalyst with other Zr-based catalysts in literatures for the conversion of furfural into furfuryl alcohol using isopropanol as hydrogen donor^a^.

Entry	Catalysts	Reaction conditions	C/%	Y/%	S/%	TOF/h^−1^	Refs.
1	Zr-CMC	90°C, 3 h	92.5	91.5	99.0	0.7	This work
2	Zr-TMSA	70°C, 5 h	93.6	89.5	95.6	0.4	Zhou et al. (2017)
3^b^	ZrPN	100°C, 15 h	93.0	90.0	96.8	0.4	Li et al. (2016b)
4	Zr-HAs	50°C, 15 h	97.4	96.9	99.0	0.1	Sha et al. (2017)
5^c^	Zr-PhyA	100°C, 2 h	99.3	99.3	100.0	0.8	Song et al. (2015b)
6	Zr-RSL (1:1)	90°C, 6 h	93.4	80.9	86.7	1.0	Hao et al. (2019)
7	Zr-SBA-15	90°C, 6 h	50.0	40.0	80.0	0.8	Iglesias et al. (2015)
8^d^	Pd/Zr-BTC	20°C, 4 h (5bar)	98.4	98.4	100.0	—	Lestari et al. (2022)
9	Zr-HPAA	150°C, 1.5 h	98.0	96.0	97.9	—	Liu et al. (2019)

a C, conversion of furfural; Y, yield of furfuryl alcohol; S, selectivity of FAL. The values of turnover frequency (TOF) were calculated by the mole of the product furfuryl alcohol/(mole of the active metals * reaction time).

b Zr-PN, organotriphosphate-zirconium hybrid.

c Zr-PhyA, Zr-phytic acid hybrid.

d The reaction uses H_2_O as hydrogen source.

### 3.4 Reusability of Zr-CMC catalysts

**FIGURE 4:**
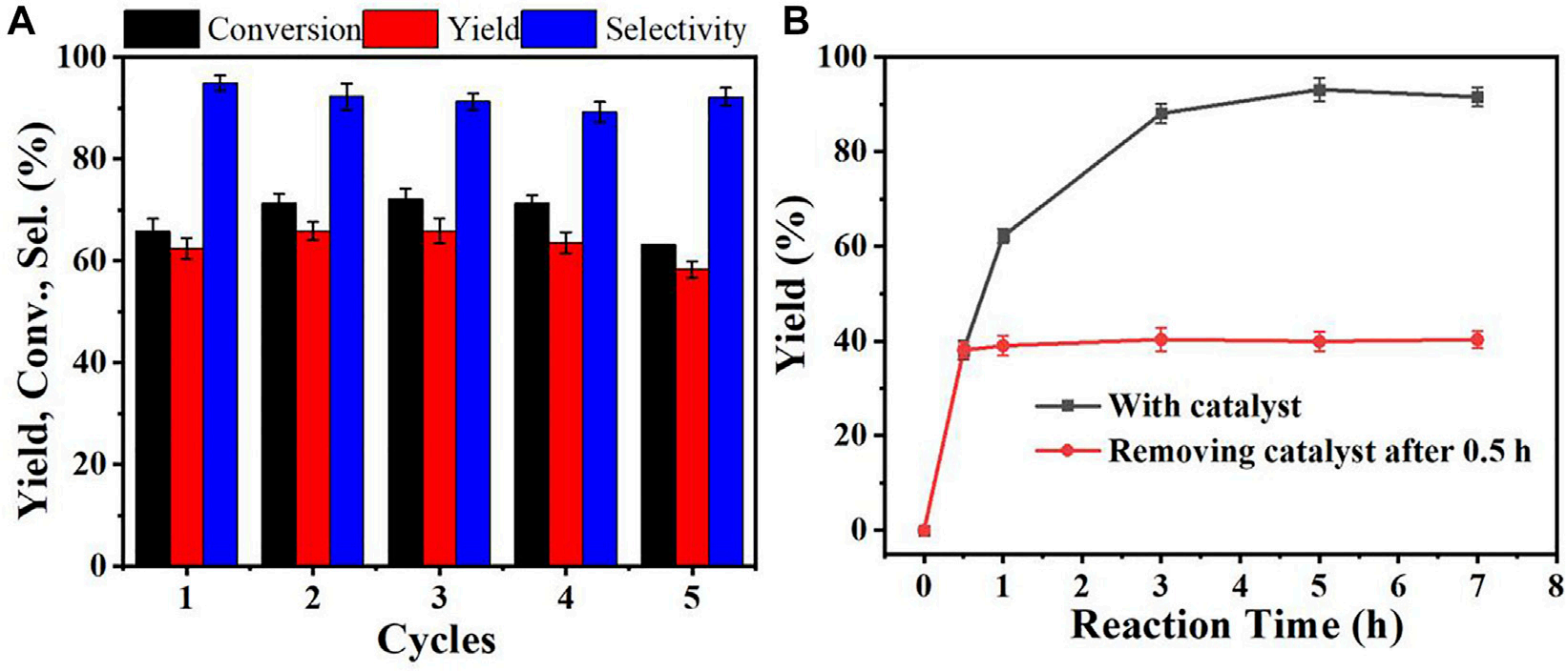
Reusability **(A)** and heterogeneity **(B)** of the prepared catalyst. Reaction conditions: Furfural 1 mmol, isopropanol 5 ml, Zr-CMC 0.2 g, reaction temperature 90°C, and reaction time 1 h (0.5 h for heterogeneity).

Reusability and heterogeneity were important for the solid heterogeneous catalyst. The reusability of Zr-CMC catalyst showed that the conversion, selectivity and product yield of the catalyst did not decrease significantly after repeated use for 5 times, indicating that the catalyst has good stability and can be reused (Figure 4A). The heterogeneity of Zr-CMC catalyst was also studied. The results showed that the reaction stopped after the catalyst was removed from the system, and no overflow of the active site of the catalyst was detected in the reaction solution, indicating that the catalyst was heterogeneous in the catalytic process (Figure 4B).

## 4 Conclusion

In summary, a route of using the sodium carboxymethyl cellulose (CMC-Na) from cellulose derivatives to construct Zr-CMC catalyst was identified. The results of SEM and TEM showed that the catalyst was irregularly granular. TG results showed that the Zr-CMC catalyst has good thermal stability at the reaction temperature. FT-IR showed that Zr^4+^ was successfully coordinated with carboxyl group in CMC-Na. The XRD results showed that the prepared catalyst was amorphous. The reaction results showed that when the mass ratio of CMC-Na to ZrOCl_2_·8H_2_O was 1: 2, the reaction activity of the obtained catalyst was the highest. Under the condition of furfural 1 mmol, reaction temperature 90°C, catalyst 0.2 g, reaction time 5 h, the conversion of furfural, the selectivity and yield of furfuryl alcohol could be reached to 92.5%, 91.5 %, and 99.0%, respectively. The heterogeneity experiment showed that the catalyst was heterogeneous during the reaction, and the activity did not change significantly when the catalyst was recycled 5 times. On the whole, the catalyst could be reused and had good stability. The proposed route in this work may find its potential applications in the field of biomass utilization with the advantages of high efficiency of the catalysts, broad applicability, and simple preparing processes.

## Data Availability

The original contributions presented in the study are included in the article/supplementary material, further inquiries can be directed to the corresponding author.
